# *In Vivo* Evaluation of Ethanolic Extract of *Zingiber officinale* Rhizomes for Its Protective Effect against Liver Cirrhosis

**DOI:** 10.1155/2013/918460

**Published:** 2013-12-12

**Authors:** Daleya Abdulaziz Bardi, Mohammed Farouq Halabi, Nor Azizan Abdullah, Elham Rouhollahi, Maryam Hajrezaie, Mahmood Ameen Abdulla

**Affiliations:** ^1^Department of Biomedical Science, Faculty of Medicine, University of Malaya, 50603 Kuala Lumpur, Malaysia; ^2^Department of Pharmacology, Faculty of Medicine, University of Malaya, 50603 Kuala Lumpur, Malaysia; ^3^Institute of Biological Science, Faculty of Science, University of Malaya, 50603 Kuala Lumpur, Malaysia

## Abstract

*Zingiber officinale* is a traditional medicine against various disorders including liver diseases.The aim of this study was to assess the hepatoprotective activity of the ethanolic extract of rhizomes of *Z. officinale* (ERZO) against thioacetamide-induced hepatotoxicity in rats. Five groups of male *Sprague Dawley* have been used. In group 1 rats received intraperitoneal (i.p.) injection of normal saline while groups 2–5 received thioacetamide (TAA, 200 mg/kg; i.p.) for induction of liver cirrhosis, thrice weekly for eight weeks. Group 3 received 50 mg/kg of silymarin. The rats in groups 4 and 5 received 250 and 500 mg/kg of ERZO (dissolved in 10% Tween), respectively. Hepatic damage was assessed grossly and microscopically for all of the groups. Results confirmed the induction of liver cirrhosis in group 2 whilst administration of silymarin or ERZO significantly reduced the impact of thioacetamide toxicity. These groups decreased fibrosis of the liver tissues. Immunohistochemistry assessment against proliferating cell nuclear antigen did not show remarkable proliferation in the ERZO-treated rats when compared with group 2. Moreover, factions of the ERZO extract were tested on Hep-G2 cells and showed antiproliferative activity (IC_50_ 38–60 **μ**g/mL). This study showed hepatoprotective effect of ERZO.

## 1. Introduction

Ginger or *Zingiber officinale* (family: Zingiberaceae) is a perennial reed-like plant with annual leafy stems. The plant is about a meter tall. The fragrant perisperm of Zingiberaceae is used as sweetmeats by the Bantu tribe [1]. Ginger is traditionally used as a common condiment for various foods and beverages such as soup, ginger ale, ginger bread, ginger snaps, parkin, ginger biscuits, and speculoos. Ginger rhizomes contain a number of pungent constituents and active ingredients [2]. The steam distillation of ginger powder is used to produce ginger oil and contains high amount of sesquiterpene hydrocarbons, predominantly zingiberene [3]. Gingerol is of the major pungent compounds in ginger and can be altered to shogaols, zingerone, and paradol [4] which takes part in several activities such as hepatoprotective [5], antiparasitic [6], antiflarial [7], antimicrobial [8], antidiabetic [9], and radioprotective [10]. Ginger also has a potential remedy against cardiovascular disease [11] and can prevent the development of morphine analgesic tolerance and physical dependence in rats [12]. The plant contains high level of total phenolic and flavonoid, responsible for its high antioxidant activities [13] (for reviews see [14, 15]).

Liver disease is still a worldwide health problem. It develops in about one-third of patients with chronic liver diseases [16] with considerable level of morbidity and mortality [17]. Liver cirrhosis is an irreversible process characterized by excess extracellular matrix (ECM) deposition in the liver accompanied with scar formation and destruction of the liver architecture [18]. During liver cirrhosis, the normal tissue is replaced with the scar tissue, which can block the blood flow of the liver. Liver cirrhosis also attenuates the liver's functions. The liver by itself is able to regenerate the damaged parts, but, during end-stage cirrhosis, the liver is not capable of renovating the damaged cells. Etiology of the liver cirrhosis has a wide spectrum including viruses, toxins, drugs, and life style [19]. Despite several efforts in drug discovery, treatment for liver cirrhosis is still a concern. In fact complementary and alternative medicine is one of the promising resources for treatment of liver cirrhosis. In traditional remedies herbal drugs have been used for the treatment of liver ailments. Many medicinal plants have been introduced with hepatoprotective potential such as *Vitex negundo* [20], *Boesenbergia rotunda* [21], *Phyllanthus niruri* [22], *Ipomoea aquatic* [23], and *Orthosiphon stamineus* [24]. The phytochemicals phenolic compounds exhibit antioxidant properties that scavenge the free radicals and ROS [25]. Antioxidant activity is important in the treatment of liver cirrhosis [26, 27]. Thioacetamide (TAA) is a hepatotoxic chemical and is widely used in induction of hepatic necrosis. Moreover, thioacetamide causes oxidative stress during its metabolism by microsomal CYP2E1. These effects together cause acute hepatitis which consequently leads to apoptosis of liver cells. Several phytochemicals have been reported in *Z. officinale* with hepatoprotective activity. For instance, in liver, cineole may inhibit CYP2E1 [28] and tocopherol and hepatocyte lipid peroxidation [29]. Inhibition of active caspase-3, capsaicin, possesses hepatoprotective effect through its antioxidant and free radical scavenging mechanisms [30]. Hepatoprotective activity of the ethanolic extract of rhizomes of* Z. officinale *(ERZO) has not been reported specifically. Thus, the present study was to assess the hepatoprotective effect of ERZO against TAA-induced liver cirrhosis in rats.

## 2. Materials and Methods

### 2.1. Chemicals

In this study we used TAA (Sigma-Aldrich, Germany) for induction of liver cirrhosis in the rats. Silymarin, a standard drug, was purchased from International Laboratory USA. Ethanol (95%; industrial graded; R&M chemical, UK) and 10% Tween-20 (Merck, Germany), concentrated formalin (38–40%; Merck, Germany), Di-natrium hydrogenphosphat (Merck, Germany), sodium dihydrogen phosphate monohydrate (Sigma-Aldrich, Germany), toluene (Merck, Germany), xylene (BDH Laboratory supplies, England), and other ordinary laboratory materials were also obtained for this experiment.

### 2.2. Preparation of the Plant Extract

Fresh rhizome of the plant was purchased from a commercial company (Ethno Resources Sdn Bhd, Selangor, Malaysia) and was identified by the voucher specimen deposited at the Herbarium of Rimba Ilmu, Institute of Science Biology, University of Malaya. The rhizome was dried and ground into fine powder using an electrical blender. Fine powder (100 g) was homogenized in ethanol (95%; 500 mL) and left in a conical flask at room temperature for 3 days. Then, the mixture was filtered through a fine muslin cloth and a filter paper (Whatman No. 1). Using the Eyela rotary evaporator (Sigma-Aldrich, USA) the extract became concentrated. The extract was then lyophilized and yielded ERZO. Tween-20 (10%) was used to dissolve the extract in the concentrations of 50 mg/mL and 100 mg/mL. In this study doses of 250 mg/kg and 500 mg/kg were considered for the oral administration of ERZO.

Liquid-liquid partitioning was also performed on the crude ERZO. Briefly, the extract was reconstituted with distilled water (150 mL × 3) to form a suspension. Then it partitioned with n-hexane (200 mL × 3), chloroform (200 mL × 3), and butanol (200 mL × 3) to obtain soluble fractions of n-hexane, chloroform, and butanol. The fractions were mixed well, inverting the whole separation funnel. The suspension was allowed to be separated overnight. For each solvent, the separation was performed thrice. The organic fractions were pooled and the same procedures were performed to yield the respective fractions. Each dried fraction was then dissolved in DMSO (0.5%) in different concentrations for the cell culture experiment.

### 2.3. Animal Experiments

The study was approved by the ethics committee for animal experimentation, Faculty of Medicine, University of Malaya, Malaysia, and the Ethic no. PM/07/05/2011/MMA (a) (R). All animals received human care according to the criteria outlined in the “Guide for the Care and Use of laboratory Animals” prepared by the National Academy of Sciences and published by the national Institute of health. The rats were provided from the Experimental Animal House, Faculty of Medicine, University of Malaya. The animals were kept at 25 ± 2°C (humidity, 50–60%) with 12 h light/dark cycle.

### 2.4. Acute Toxicity Test

The toxicity of the ERZO was evaluated in *Sprague Dawley* rats. The animals were treated with two distinct doses of ERZO. The animals were given standard rat pellets and tap water *ad libitum*. Thirty-six rats (18 males and 18 females), weighed 150–180 g, were assigned into 3 groups named control group (Tween-20 10% w/v; 5 mL/kg), low dose group (ERZO, 2 g/kg), and high dose group (ERZO, 5 g/kg). Prior to the dosing, the rats fasted (food but water) overnight. Food was withheld for a further 3 to 4 hours after dosing. Each group received their respective administration, orally. Then, the animals were observed high frequently for 48 h for any sign of abnormality. The rats were monitored for 14 days for any sign of toxicity. The animals were sacrificed on the 15th day. Histological, hematological, and serum biochemical parameters were also assessed [31].

### 2.5. Induction of Liver Injury

Thirty male SD rats (6–8 weeks old; weighted 150–180 g) were obtained from the Experimental Animal House, Faculty of Medicine, University of Malaya. The rats were randomly divided into 5 groups of 6 rats and kept individually in a cage with wide-mesh wire bottom (i.e., to prevent coprophagia during the experiment). Group 1 received i.p. injection of normal saline, thrice weekly, and oral admonition of distilled water (5 mL/kg), daily. Groups 2–5 were injected with 200 mg/kg TAA (i.p.), thrice weekly as previously described [32]. Group 2 received oral administraion of distilled water (5 mL/kg). Group 5 received silymarin (50 mg/kg) as standard drug. Groups 4 and 5 were fed daily with 250 mg/kg and 500 mg/kg of ERZO. The animals were given water ad libitum. The body weights of the animals were recorded weekly. The duration of the experiment was 8 weeks, according to the previous published work [24]. The rats fasted for 24 h after receiving their respective treatments. Then the animals were euthanized with ketamine (30 mg/kg) and xylazil (3 mg/kg) and diethyl ether inhalation. For each rat, the abdomen and thoracic cavities were opened. The internal organs were checked to be assured that the other organs appear intact, microscopically. The liver was washed with ice-cold normal saline and phosphate buffered saline (PBS; pH 7.4). The organ was carefully dissected and assessed grossly. The liver's weight was recorded for each animal. The tissues were preceded for immunohistology evaluations and antioxidant activities. Blood was sampled through jugular vein to assess the liver's function.

### 2.6. Biochemical Parameters for Liver Function

Blood analysis was performed at the Clinical Diagnosis Laboratory of University of Malaya Hospital. The main biochemical parameters to assess the liver function were aspartate aminotransferase (AST), alanine aminotransferase (ALT), alkaline phosphatase (ALP), gamma glutamyl transferase (GGT), globulin, conjugated bilirubin, total bilirubin, albumin, and total protein.

### 2.7. Histopathology of Liver Tissues

Liver tissues were fixed in 10% formalin. The fixed tissues were processed using an automated tissue processing machine (Leica, Germany) and were embedded in paraffin (Leica, Germany). Sections of 5 *μ*m thickness were prepared for each liver tissue. The sections were processed for hematoxylin and eosin (H&E) and Masson's Trichrome (MT) staining. Another set of sections were prepared for immunohistochemistry study of the tissues and mitotic indexing. The liver tissues were further assessed for histopathological examination in a blinded fashion.

### 2.8. Immunohistochemistry and Mitotic Index

The sections were heated at 60°C for 60 min in an oven (Venticell, MMM, Einrichtungen, Germany). Then the tissues were deparaffinized with xylene. The dehydrating step was performed with graded alcohols (absolute alcohol, 95% and 70% alcohol). Antigen retrieval was performed with 10 mM sodium citrate buffer boiling in a microwave (Sanyo, Super Showe wave, Japan). The sections were placed in TBS contained Tween-20 (0.05%). The staining steps were performed according to the manufacturer's instructions (DakoCytomation, USA). In short, endogenous peroxidase was quenched by peroxidase blocking solution. Then the sections were incubated with proliferating cell nuclear antigen (PCNA; 1 : 200), a biotinylated primary antibodies, for 15 min. Then, streptavidin-HRP was added and incubated for 15 min. The sections were incubated with diaminobenzidine-substrate chromagen DAB for 5 min. The sections were then dipped in weak ammonia (0.037 M/L). Under light microscopy, PCNA positive tissues were stained brown with blue background.

Cell proliferation was assessed though counting the number of mitotic cells per high-power field (HPF) at the magnification of 100x. For each slide 10 randomly selected fields were counted. The mitotic index (MI) was defined as the number of mitotic cells per 1,000 hepatocytes in paraffin-embedded liver samples stained with H&E [33].

### 2.9. Superoxide Dismutase and Malondialdehyde in Liver Tissue

The tissue samples were homogenized in cold 20 mM HEPES buffer (1 mM EGTA, 210 mM mannitol, and 70 mM sucrose; pH 7.2) using tephlon homogenizer (Polytron, Heidolph RZR 1, Germany). The cell debris was separated by centrifugation (Heraeus, Germany) at 1,500 g for 5 min (4°C). The supernatants were used for the estimation of SOD activity (Cayman Chemical Company, USA). The MDA level was also measured by thiobarbituric acid (TBARS) according to the manufacturer protocol (Cayman Chemical Company, USA).

### 2.10. Cell Culture and Cytotoxicity of ERZO's Fractions

There were four different fractions, ZC, ZX, ZB, and ZW, representing chloroform, n-hexane, butanol, and aqueous fractions of *Z. officinale*, respectively. A human liver carcinoma cell line (Hep-G2) was obtained from American Type Culture Collection (ATCC, USA). The 3-(4, 5-dimethylthiazol-2-yl)-2, 5-diphenyl tetrazolium bromide (MTT assay) was used to assess the number of viable cells which has been adapted to measure the growth of cells *in vitro*. According to modified protocol [34], the assay was adapted to 96-well cell culture plates. Approximately, 5000 cells per well were seeded one day before the experiment. Cell lines were cultured in RPMI-1640 growth medium, supplemented with ERZO's fractions at different concentrations (3–200 *μ*g/mL), 10% (v/v) sterile fetal bovine serum (FBS, PAA Lab, Austria), 100 mg/mL streptomycin and 100 U/mL penicillin (PAA Lab, Austria), and 50 mg/mL fungizone (Sigma Aldrich). The cultures were incubated in 5% CO_2_ incubator at 37°C in a humidified atmosphere. The cells were harvested by detaching the cells from the culture flask using 1-2 mL of trypsin after the flask get confluent enough with the cells. The harvested cells were transferred aseptically into 50 mL sterile falcon tube and washed with physiological buffer (pH 7.2) under spinning at 1200 rpm for 2 minutes. The supernatant was discarded, and the cells pellet was mixed with 1 mL of sterile media and was mixed to form a cell suspension. Harvested cells were seeded into a 96-well culture plates at 100 *μ*L/well and allowed to adhere overnight. ERZO's fractions were predissolved in dimethyl sulphoxide (DMSO) and diluted to different concentration spanning from 3–200 *μ*g/mL. Blank DMSO was used as a control. Cells were incubated with the samples (three wells on a plate for each concentration) for 24 to 72 h. Thereafter, 10 *μ*L of MTT (5 mg/mL) (Sigma) was added to each well and the plates were incubated at 37°C for 4 h. The media were then gently removed, and about 200 mL of DMSO was added to dissolve the formazan crystals. The MTT formazan production was quantified spectrophotometrically at 570 nm using a microplate reader (GF-M3000) for acidified isopropanol and at 555 nm for DMSO. OD reading was referenced to 700 nm to eliminate the background effect. The percentage cell viability was calculated according to the following equation:

(1) $$\begin{array}{c} \text{Cell}\;\text{viability}\%=\left(\frac{{\text{Abs}}_{570}\;\text{treated}}{{\text{Abs}}_{570}\;\text{untreated}}\right)\times 100. \end{array}$$

The IC_50_ value was calculated using the line graph which was drawn with % inhibition and sample concentration.

### 2.11. Statistical Analysis

In this study statistical analysis was done using one-way analysis of variance (ANOVA) followed by Bonferroni's post hoc test (SPSS ver. 20; SPSS Inc., USA). The *P* value less than 0.05 was considered significant.

## 3. Results

### 3.1. Acute Toxicity Tests

The animals pretreated with ERZO (2 g/kg and 5 g/kg) remained alive and did not manifest any significant visible sign of toxicity within the experimental period. There was no clinical and behavioral abnormality. Histological assessment also confirmed that the extract is safe in doses less than 5 g/kg within 15 days.

### 3.2. Liver Injury and the Effects of ERZO on Liver

**Figure 1:**
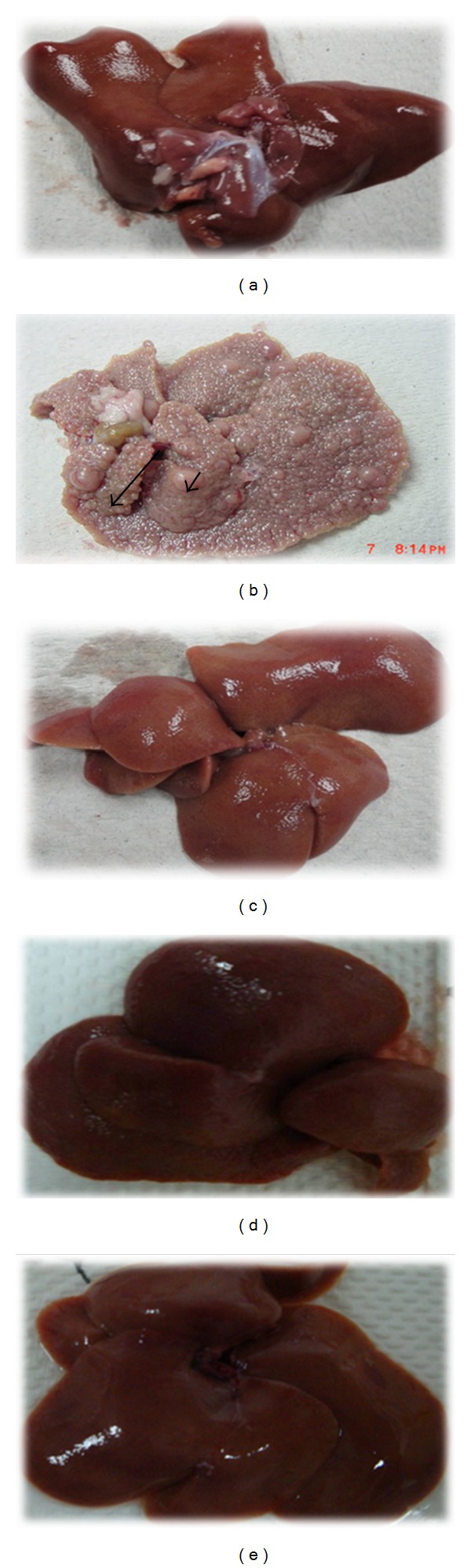
Gross morphology shows the effects of ERZO on thioacetamide TAA induced liver damage in rats. (a) Normal control group shows a regular and smooth surface. (b) Animals treated with TAA show many micronodules (arrowhead) and macronodules in the liver (arrow). (c) Animals treated with TAA + silymarin showing normal smooth surface. (d) Animals treated with TAA + ERZO 250 mg/kg and (e) animals treated with TAA + ERZO 500 mg/kg. Both low dose and high dose of ERZO were having normal smooth surface and nearly preserve the liver normal anatomical shape and appearance.

Figure 1 shows gross appearance of the liver for each group. Liver in all of the groups except group 2 possessed generally smooth surfaces without significant irregularities or sign of nodules.  Table 1 lists the body weight and the liver weight for each rat during the experiment. Group 1 demonstrated a normal growth pattern for healthy rats. Administration of TAA to group 2 lowered the body weight dramatically. The administration of ERZO (250 mg/kg and 500 mg/kg) was comparable with the effects observed in group 3 (silymarin treated group).

### 3.3. Biomedical Parameter for Liver Function

According to Tables 2 and 3, group 2 exhibits the highest levels of ALP, ALT, AST, GGT, globulin, total bilirubin, and conjugated bilirubin but albumin and total protein were the lowest among the groups. ERZO (250 mg/kg) showed that the level of ALP, ALT, AST, GGT, globulin, total bilirubin, and conjugated bilirubin were reduced significantly, whilst albumin content was significantly elevated. In these groups, ERZO could significantly restore the biochemical parameters to the levels comparable with silymarin (the standard drug).

### 3.4. Histopathological Study

**Figure 2:**
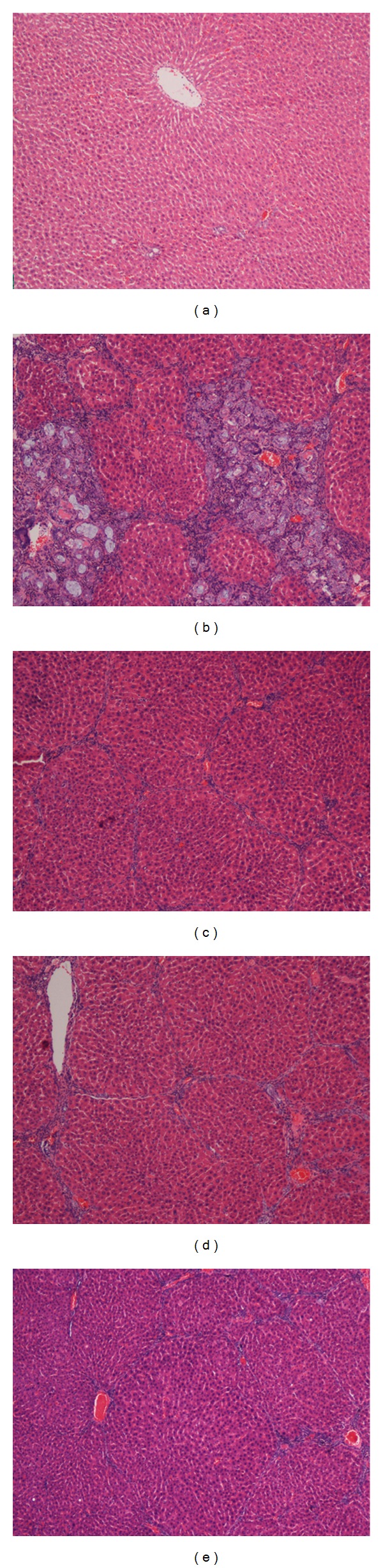
Micrograph presents the histopathological sections of the livers taken from rats in different experimental groups. (a) Normal histological structure and architecture were seen in livers of the normal control group. (b) Severe structural damage and formation of pseudolobules with thick fibrotic septa with proliferation of bile duct and centrilobular necrosis were present in the liver of the TAA control group. (c) Mild inflammation but no fibrotic septa were depicted in the liver of the hepatoprotective rat treated with TAA + silymarin. (d) Partially preserved hepatocyte and architecture with small area of necrosis and narrow fibrotic septa existed in the liver of the rat treated with TAA + 250 mg/kg of the ERZO. (e) Partially preserved hepatocyte and architecture with small areas of mild necrosis were observed in the liver of the rat treated with TAA + 500 mg/kg of the ERZO (H&E stain original magnification 10x).

A histological image is shown in Figure 2. Regular cellular architecture with distinct normal plates of hepatocytes separated by sinusoidal capillaries and central vein was remarkable in group 1 (Figure 2(a)). In TAA control group (group 2), the liver was enlarged with numerous micro- and macronodules accompanied with disrupted cellular architecture due to the presence of regenerating nodules. The liver was sectioned by fibrous septa extending from the central vein to portal triad. The liver in group 2 showed extensive hepatic damage with necrosis and severe proliferation of bile duct. Moreover, there were microvesicular and macrovesicular centrilobular type fatty changes. Intense inflammation consisting of granulocytes and mononuclear cells around the central vein and in portal areas and congestion appeared to be significant. The livers in groups 3–5 showed a relative protection against the hepatic lesions induced by TAA. There was less disruption of the hepatic cellular structure with mild fibrotic septa. Lymphocyte infiltration in these groups was not significant. They also showed occupied region of the liver by regenerative parenchyma nodules surrounded by septa of fibrous tissue with a remarkable increase in fat storing cells, Kupffer cells, and bile ductules (Figures 2(c), 2(d), and 2(e)).

**Figure 3:**
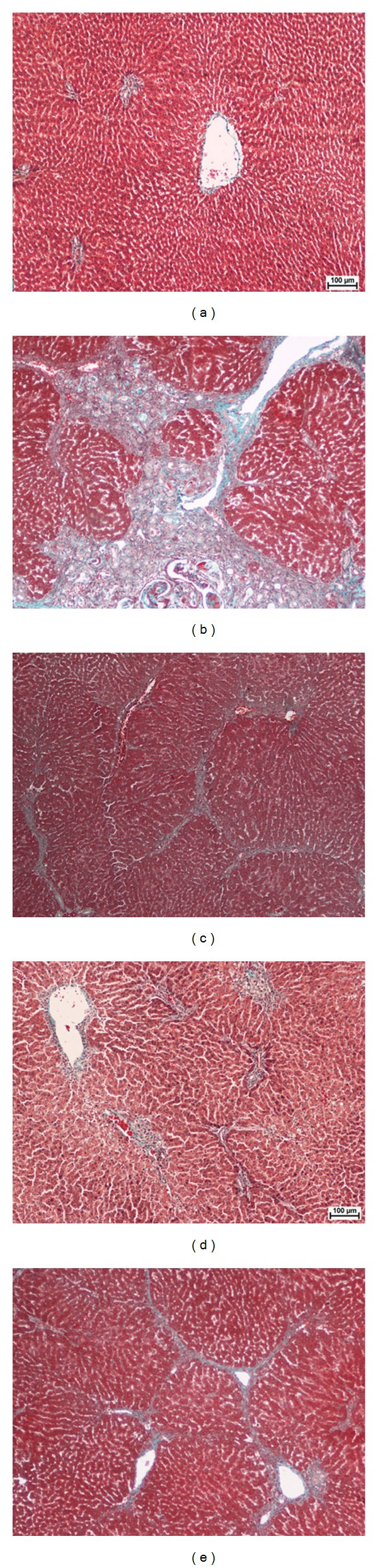
Photomicrograph shows histopathological sections of the livers sampled from different experimental groups. (a) Normal control group shows normal liver architecture. (b) TAA control group shows proliferation of bile duct, dens fibrous septa, and collagen fibers. (c) Rat treated with TAA + silymarin shows minimal fibrous septa and collagen fibers. (d) Rat treated with TAA + 250 mg/kg of the ERZO shows minimal fibrous septa and irregular regenerating nodules. (e) Rat treated with TAA + 500 mg/kg of ERZO shows minimal fibrous septa and collagen fibers. Masson's Trichrome stain (original magnification 10x).

Masson's trichrome staining was performed to evaluate the degree of fibrosis. As shown in Figure 3(a), liver section from group 1 had no collagen deposition. Group 2 showed proliferation of bile duct with dense fibrous septa and increased deposition of collagen fibers around the congested central vein, which indicate a severe fibrosis (Figure 3(b)). The rat treated with 250 mg/kg and 500 mg/kg of ERZO showed less fibrous septa and irregular regenerating nodules (Figures 3(d) and 3(e)). The collagen deposition patterns appeared comparable among groups 3–5. These observations confirmed the hepatoprotection activity of ERZO.

### 3.5. Regulation of PCNA and Mitotic Index

**Figure 4:**
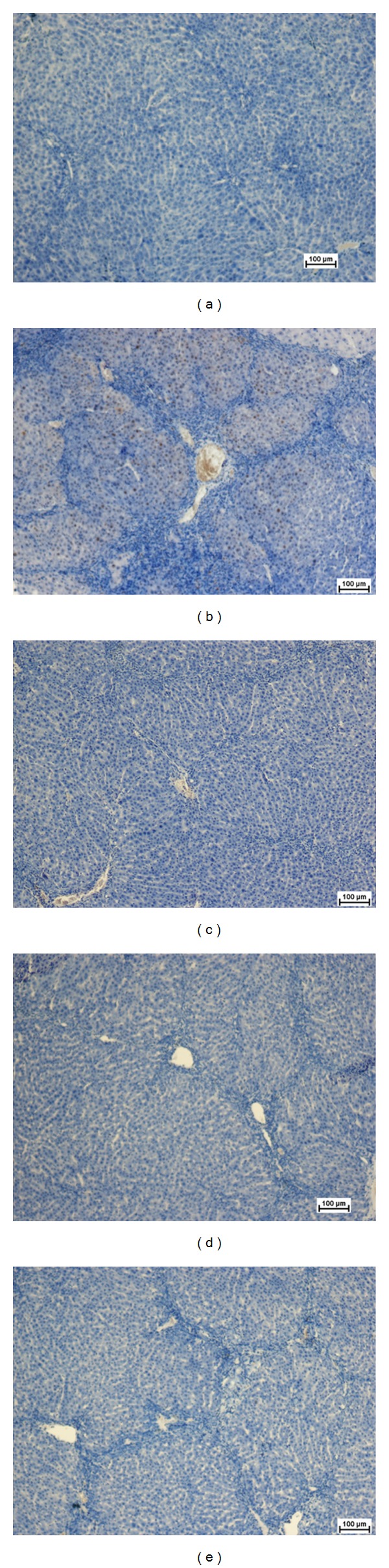
Photomicrograph shows histopathological sections of the livers sampled from different experimental groups using an anti-PCNA antibody. (a) Normal control group was stained without adding the primary antibody and shows normal liver architecture with no signs of PCNA expression. (b) TAA control group showed many hepatocytes nuclei positive for PCNA monoclonal antibody. (c) TAA + silymarin treated rats with no expression of PCNA in hepatocytes. (d) TAA + 250 mg/kg of ERZO and (e) TAA + 500 mg/kg of ERZO group showed nearly normal liver architecture with no signs of PCNA expression (immunohistochemistry, 10x).

Using immunohistochemical staining against PCNA and mitotic index, the proliferating cells were highlighted in the liver tissue sections (Figure 4 and Table 4). Normal hepatocytes as appeared in group 1 did not have PCNA positive cells. Moreover, this group did not possess significant number of mitotic cells. Similarly, group 3 had no sign of PCNA staining. In comparison, group 2 showed upregulation of PCNA as a proliferative factor for the renovation of the damage tissues caused by TAA. Administration of ERZO significantly declined the mitotic index with low level of PCNA.

### 3.6. SOD and MDA Content of Liver Homogenate

**Figure 5:**
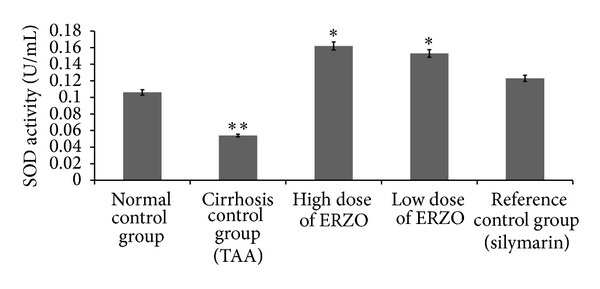
Effect of ERZO on SOD level in the liver tissue. Data are expressed as mean ± SEM. Means among groups (*n* = 6 rate/group) show significant difference, *P < 0.05 compared to TAA control group, and **P < 0.05 compared to normal control group.

**Figure 6:**
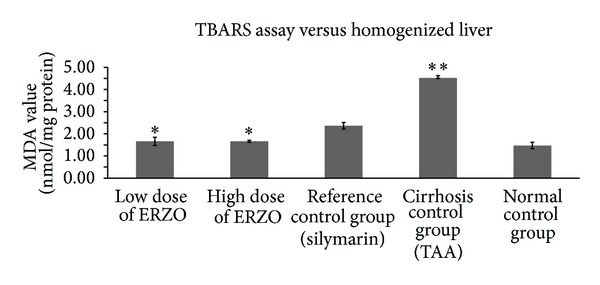
Effect of ERZO on MDA level in the liver tissue. Data are expressed as mean ± SEM. Means among groups (*n* = 6 rate/group) show significant difference, *P < 0.05 compared to TAA control group, and **P < 0.05 compared to normal control group.

The antioxidant activity for group 2 showed that SOD was significantly reduced when compared with group 1. The treated groups with ERZO (250 mg/kg and 500 mg/kg) or silymarin restored the SOD level significantly (Figure 5). MDA level of liver homogenate was significantly high in group 2 but administration of ERZO considerably lowered the level of MDA. These results were comparable with the silymarin-treated group (Figure 6).

### 3.7. Cytotoxicity Effect of ERZO's Fractions on Hep-G2

*In vitro* evaluation of ERZO's fractions against human liver carcinoma cell line is presented in Table 5. It is observed that ZX fraction at low concentration <50 mg/mL has the highest inhibition activity with corresponding 59% inhibition and IC_50_ of 38 *μ*g/mL. However, beyond 50 *μ*g/mL, ZB fraction showed the lowest efficacy with corresponding 67% inhibition and IC_50_ of 60 *μ*g/mL. In comparison to ZB fraction, ZC fraction was observed to be more potent on Hep-G2 cell line achieving a maximum inhibition of about 92% and IC_50_ of 42 *μ*g/mL. The percentage inhibition appeared to continue to increase with increasing concentration up to 200 *μ*g/mL. However, the aqueous fraction ZW performance was found to have an IC_50_ > 200 *μ*g/mL.

## 4. Discussion

Several models have been introduced in induction of hepatic injuries [35]. TAA, a most common agent used for induction of liver cirrhosis in animal studies, converts into a toxic reactive metabolite named N-acetylp-benzoquinone imine (NAPBI) and halogenates free radical in hepatic cytochrome p450 [36]. A single dose of TAA causes centrilobular necrosis and subsequent regenerative response in animals [37]. Oral administration of TAA to rodents causes apoptosis and necrosis. In fact, thioacetamide can cause liver cirrhosis and hepatocarcinoma [38, 39], hepatocellular carcinomas, hepatocellular neoplasms, bile duct, and cholangiocellular neoplasms [34]. TAA is specific for the liver and regiospecific for the perivenous hepatocytes with a long window period between necrogenic effects and liver failure [37]. In this study, acute toxicity test showed that ERZO was not toxic <5 mg/kg and did not cause any sign of toxicity or mortality within 14 days of the experiment. This observation was in accordance with the previous studies on *Z. officinale* [40, 41]. Rats in group 2 showed hepatomegaly. The increased liver weight/body weight ratio in TAA-treated animal was due to the accumulation of fat and degeneration in the liver. This result was in agreement with a previous report on the increased liver weight/body weight ratio [42]. Our result showed that ERZO could significantly accelerate the recovery of the liver damage. Liver cirrhosis caused hepatomegaly but in this experiment treatment with ERZO significantly prevents the effect of TAA as previously described [24]. The reduction of body weight seen in ERZO treatments might be due to the reduction of hyperlipidemic [43].

TAA enhanced the levels of serum biochemical parameters such as globulin, total bilirubin, and conjugated bilirubin but lowered total protein and albumin as previously reported [44]. The serum biochemical level for ALT, AST, and ALP is in accordance with the extent of liver damage [45]. These results confirmed previous works [41, 44]. The elevation in AST and ALT reflects hepatocellular injury while ALP is linked to the GGT elevation [46]. ERZO reduced the level of ALP as previously reported by Ajith et al. 2007 [47].

Histological evaluation showed protective effect of ERZO against TAA-induced liver cirrhosis. TAA by its nature induces liver cirrhosis in rats. A normal liver has a regular and smooth surface, but in liver cirrhosis it appeared rough and nodular with formations of micronodules and macronodules. In the histopathology evaluation, severe structural damage, formation of irregular pseudolobules with dense fibrotic septa, and proliferation of bile ducts in presence of centrilobular and inflammatory cells were noticeable. The treatment with silymarin and ERZO (in both doses) was considerable. ERZO could enhance reconstitution of liver structure from cirrhosis. The protection level of ERZO was dosedependent as the treatment of 500 mg/kg of ERZO had more protective effect. Moreover, a remarkable reduction in liver fibrosis was observed in ERZO (especially at 500 mg/kg), as previously confirmed [44, 48]. As Masson's trichrome staining showed, minimal reduction in collagen synthesis was seen in ERZO-treated groups. In a healthy liver, no significant collagen deposition was observed. The cytoplasm of the cells appeared clear, with dilated central veins [49, 50]. Liver cirrhosis induced by TAA caused severe fibrosis especially around bile ducts where dense fibrous septa and high deposition of collagen fibers with distorted nuclei and extensively vacuolated cytoplasm were considerable. The standard drug, silymarin, reduced collagen deposition and the cells architecture was resembled. Comparably, ERZO showed a moderate deposition of collagen fibers, especially in the dose of 500 mg/kg. Our results confirmed previous reports on the reduction of necrosis and fibrosis in hepatoxic liver cells in response to phytochemical treatments [49, 50].

The inhibitory effect of ERZO on hepatic stellate cell activation was confirmed by Lee et al. 2011 [51].

PCNA, also known as cyclin, is a nuclear protein synthesized in G_1_/S-phase of the cell cycle and is related to cell proliferative activity [52–54]. PCNA is polymerase S accessory protein, essential for cellular DNA synthesis [54]. Liver cirrhosis showed upregulation of PCNA, indicating extensive proliferation for the replacement of the damaged tissues. Treatment with ERZO or silymarin significantly reduced the expression of PCNA as previously reported [55]. The antioxidant test for SOD for the treated rats with ERZO showed high level of activity in comparison with the TAA control group (group 2). The SOD activity appeared similar in both groups treated with ERZO or silymarin which confirmed the previously published work [47]. Reactive oxygen species and nitric oxide are responsible for the induction of hepatocytes apoptosis [56]. Administration of ERZO significantly reduced MDA activity as previously observed in adriamycin-induced model [57].

On evaluating the antiapoptotic efficacy, Hep-G2 cell line was used in this analysis. n-Hexan fraction of ERZO proved to be more potent on Hep-G2 cell line. On the other hand, chloroform fraction of ERZO showed a moderate potency. Butanol fraction of ERZO showed lower efficacy. However, water fraction of ERZO was observed to show the lowest inhibitory activity. In general, the observed efficacy could probably be due to the phytochemical constituents of this plant. The antioxidant action of *Z. officinale* has been proposed as one of the major possible mechanisms for the protective actions of the plant against toxicity. It has been shown that [6]-gingerol is endowed with strong antioxidant action both *in vivo* and *in vitro*, in addition to strong anti-inflammatory and antiapoptotic actions. Pretreatment with [6]-gingerol reduced UVB-induced intracellular reactive oxygen species levels, activation of caspases 3, 8, and 9, and Fas expression. It also reduced UVB-induced expression and transactivation of COX-2 [58]. The hepatoprotective effect of ERZO is due to its antioxidant potency [13] dominated by monoterpenoids [59]. This could be due to the fact that flavonoids have the ability to inhibit lipid peroxidation [5]. These data suggested that ERZO is a very effective agent for the prevention of liver cirrhosis.

## 5. Conclusion

In the present study, the hepatoprotective activity of ERZO was explored *in vitro *and *in vivo*. Histology, ERZO slowed down liver fibrosis progression and prevents the generation of free radical induced by TAA, which provide an insight into the mechanism of its biological action. According to these data, *Z. officinale *ingestion is safe in humans and might be a promising hepatoprotective agent.

## Supplementary Material

Serum biochemical parameters and microscopic evaluation for the acute toxicity test showed no sign of toxicity, nephrotoxicity and/or hepatotoxicity.

## Figures and Tables

**Table 1:** Effects of ERZO on body weight, liver weight, and liver index.

Animal groups	Body weight (gm)	Liver weight (gm)	Liver index (LW/BW %)
Normal control	347 ± 6.65^b^	10.33 ± 1.15	2.95 ± 0.22^b^
TAA control	172 ± 6.47^a^	12.61 ± 0.41	7.38 ± 0.36^a^
HD 500 mg/kg	217 ± 3.44^ab^	10.14 ± 0.53^b^	4.66 ± 0.21^ab^
LD 250 mg/kg	231 ± 8.76^ab^	11.78 ± 0.18	5.11 ± 0.19^ab^
Silymarin 50 mg/kg	374 ± 8.16^b^	10.66 ± 1.69	2.99 ± 0.32^b^

Data are expressed as mean ± SEM. Means among groups (*n* = 6 rats/group) show significant difference.

^b^ *P* < 0.05 compared to TAA control group, and ^a^ *P* < 0.05 compared to normal control group.

**Table 2:** Effect of TAA, silymarin, and ERZO on biochemical parameters in the serum of experimental rats.

Animal groups	T. protein (g/L)	Albumin (g/L)	Globulin (g/L)	T. bilirubin (umol/L)	Conjugated bilirubin (umol/L)
Normal control	68.67 ± 1.4^b^	12.83 ± 1.70^b^	54.50 ± 0.54^b^	2.66 ± 0.5^b^	1 ± 0.00^b^
TAA control	60.83 ± 0.47^a^	7.83 ± 0.40^a^	68.66 ± 3.44^a^	9 ± 0.21^a^	5.3 ± 0.00^a^
HD 500 mg/kg	67.66 ± 0.41	12 ± 0.63^b^	54.16 ± 1.72^b^	5 ± 1.26^a^	3 ± 0.22^ab^
LD 250 mg/kg	65.33 ± 0.33	11.50 ± 1.04^b^	51.50 ± 2.85^b^	7.33 ± 1.21^a^	3.5 ± 0.61^a^
Silymarin 50 mg/kg	67.33 ± 0.95^b^	11.83 ± 1.83^b^	54.33 ± 3.61^b^	5.66 ± 1.86	3 ± 0.36^a^

Data are expressed as mean ± SEM. Means among groups (*n* = 6 rats/group) show significant difference.

^b^ *P* < 0.05 compared to TAA control group, and ^a^ *P* < 0.05 compared to normal control group.

**Table 3:** Effects of pretreatment with TAA, silymarin, and ERZO on serum liver biomarkers of experimental rats.

Animal groups	ALP (IU/L)	ALT (IU/L)	AST (IU/L)	GGT (IU/L)
Normal control	100.67 ± 4.96^b^	64 ± 1.26^b^	174.5 ± 8.11^b^	5 ± 0.00^b^
TAA control	243.83 ± 18.33^a^	209.83 ± 5.23^a^	322.16 ± 6.17^a^	12 ± 0.00^a^
HD 500 mg/kg	119.67 ± 3.72^ab^	63.83 ± 7.25^b^	180.83 ± 1.94^b^	6.67 ± 0.55^ab^
LD 250 mg/kg	218.83 ± 16.58^a^	82.5 ± 3.09^ab^	227.83 ± 4.62^b^	6.83 ± 0.16^b^
Silymarin 50 mg/kg	132.66 ± 10.44^b^	70.3 ± 4.92^b^	184.33 ± 7.9^b^	7 ± 0.00^ab^

Data are expressed as mean ± SEM. Means among groups (*n* = 6 rats/group) show significant difference.

^b^ *P* < 0.05 compared to TAA control group, and ^a^ *P* < 0.05 compared to normal control group.

**Table 4:** Effects of pretreatment with TAA, silymarin, and ERZO on mitotic index.

Animal groups	Mitotic index
Normal control	0
TAA control	2.8 ± 1.15^a^
HD 500 mg/kg	0.5 ± 0.16
LD 250 mg/kg	0.7 ± 0.08
Silymarin 50 mg/kg	0.4 ± 0.02

Data are expressed as mean ± SEM. Means among groups (*n* = 6 rats/group) show significant difference; ^a^ *P* < 0.05 compared to normal control group.

**Table 5:** Antiapoptotic efficacy of ERZO's fractions by MTT assay on Hep-G2 cell line.

ERZO fractions	IC50 (*µ*g/mL)
ZX	38
ZC	42
ZB	60
ZW	>200
