# Supplementary material for: In Vivo Evaluation of Ethanolic Extract of Zingiber officinale Rhizomes for Its Protective Effect against Liver Cirrhosis
Source: Biomed Res Int. 2013 Dec 12;2013:918460. doi: 10.1155/2013/918460 (PMC3874366; doi:10.1155/2013/918460)
Supplement: Supplementary file 1 — Serum biochemical parameters and microscopic evaluation for the acute toxicity test showed no sign of toxicity, nephrotoxicity and/or hepatotoxicity. [file 918460.f1.pdf]

Table 1: Effect of ERZO on liver function biochemical parameters in rats

	Liver function tests					
**Dosage**	**ALT U/L**	**ALP U/L**	**AST U/L**	**T. Bil Umol/L**	**Albumin g/L**	**T. protein g/L**
**Male**						
Vehicle	44.12 + 6.17	142.55 ± 18.38	193.51 ± 22.51	1.84± 0.77	30.22 ± 7.05	56.50± 8.24
2,000 mg/kg	42.88 + 6.07	140.81 ± 15.77	183.50 ± 20.27	2.00± 0.85	28.75 ± 6.35	54.88 ± 9.05
5,000 mg/kg	43.65 + 5.28	139.79 ± 16.24	195.11 ± 21.67	2.07 ± 0.71	31.52 ± 7.78	51.11 ± 8.57
**Female**						
Vehicle	46.15 + 5.36	144.38 ± 15.73	197.65 ± 21.26	2.01 ± 0.51	35.35± 9.77	57.55 ± 8.83
2,000 mg/kg	42.91 + 6.13	140.45 ± 15.05	191.87 ± 20.66	1.89± 0.68	32.37 ±8.03	55.26 ± 10.03
5,000 mg/kg	44.08 + 6.44	146.37 ± 14.85	188.30 ± 22.13	2.15 ± 0.85	37.98 ± 9.19	53.25 ± 8.23

All values have been expressed as mean ±S.E.M. Means are significantly different at $p < 0.05$

Table 2: Effect of ERZO on renal function biochemical parameters in rats

	Kidney function tests					
**Dosage**	**Calcium**	**Createnin**	**Urea**	**Potassium**	**Sodium**	**Chloride**
**Male**						
Vehicle	2.29 ±0.56	61.55 ± 6.17	5.55 ± 0.89	4.03 ± 0.83	152.01± 10.24	112.32± 8.05
2,000 mg/kg	2.18 ± 0.54	63.36 ± 5.11	5.45 ± 0.77	4.33 ± 0.95	150.24±9.837	118.19± 9.64
5,000 mg/kg	2.44 ± 0.37	68.08 ± 5.86	5.20 ± 0.69	4.25 ± 0.80	148.65±9.55	111.33 ± 10.03
**Female**						
Vehicle	2.55± 0.58	65.55± 5.48	5.59 ± 0.84	4.17 ± 0.92	150.45± 12.05	115.51±10.48
2,000 mg/kg	2.46 ± 0.73	63.28 ±7.13	5.85 ± 0.88	4.55 ±0.80	145.85± 11.13	117.21±11.11
5,000 mg/kg	2.35 ± 0.51	70.11 ± 5.06	5.72 ± 0.56	4.48 ± 0.78	148.78±11.67	122.98±10.15

All values have been expressed as mean ±S.E.M. Means are significantly different at $p < 0.05$

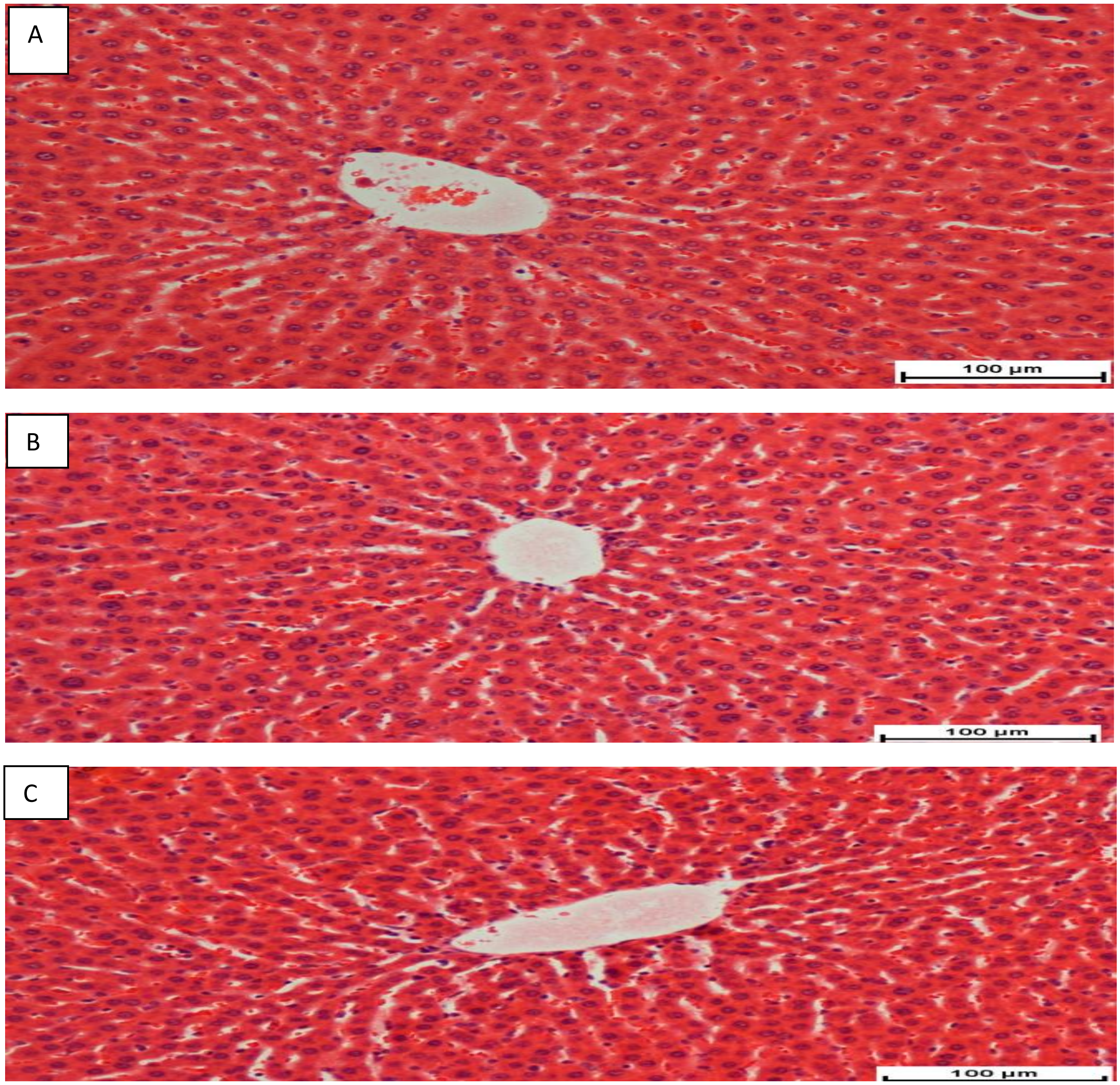

Figure 1: Histological section of liver in: (A) animal treated with vehicle (10% Tween 20), (B) animal treated with (ERZO, 2 g/kg) and (C) animal treated with (ERZO, 5 g/kg), respectfully, showed normal structural appearance of liver parenchyma (H&E stain 20 x).

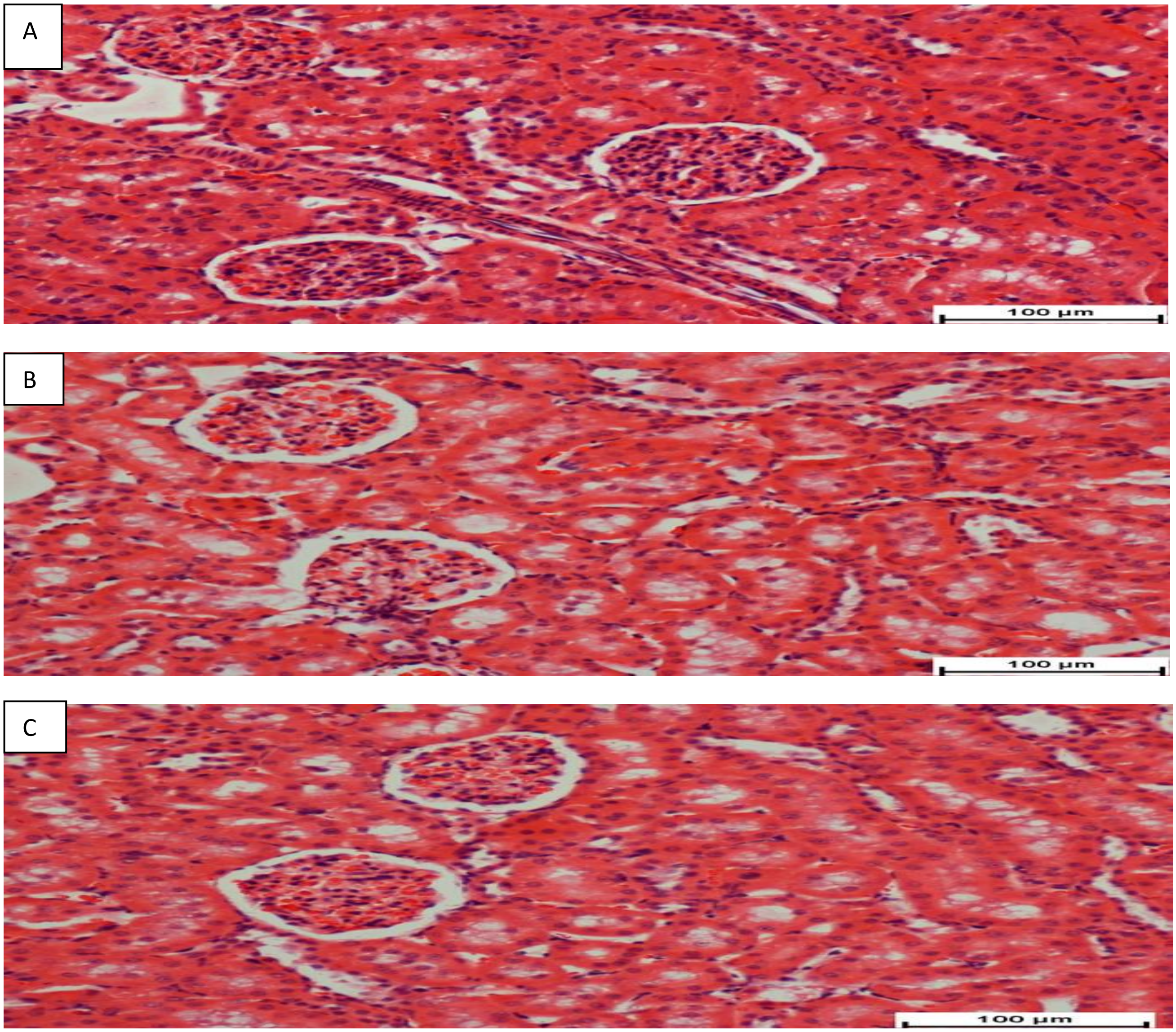

Figure: 2: Histological section of kidney in: (A) animal treated with vehicle (10% Tween 20), (B) animal treated with (ERZO, 2 g/kg) and (C) animal treated with (ERZO, 5 g/kg), respectfully, showed normal structural appearance of kidney parenchyma (H&E stain 20 x).
